# A Computationally Efficient Finite Element Pedestrian Model for Head Safety: Development and Validation

**DOI:** 10.1155/2019/4930803

**Published:** 2019-07-24

**Authors:** Guibing Li, Zheng Tan, Xiaojiang Lv, Lihai Ren

**Affiliations:** ^1^School of Mechanical Engineering, Hunan University of Science and Technology, Xiangtan 411201, China; ^2^State Key Laboratory of Advanced Design and Manufacturing for Vehicle Body, Hunan University, Changsha 410082, China; ^3^Zhejiang Key Laboratory of Automobile Safety Technology, Geely Automobile Research Institute, Ningbo 315336, China; ^4^Key Laboratory of Advanced Manufacturing Technology for Automobile Parts, Ministry of Education, Chongqing University of Technology, Chongqing 400054, China

## Abstract

Head injuries are often fatal or of sufficient severity to pedestrians in vehicle crashes. Finite element (FE) simulation provides an effective approach to understand pedestrian head injury mechanisms in vehicle crashes. However, studies of pedestrian head safety considering full human body response and a broad range of impact scenarios are still scarce due to the long computing time of the current FE human body models in expensive simulations. Therefore, the purpose of this study is to develop and validate a computationally efficient FE pedestrian model for future studies of pedestrian head safety. Firstly, a FE pedestrian model with a relatively small number of elements (432,694 elements) was developed in the current study. This pedestrian model was then validated at both segment and full body levels against cadaver test data. The simulation results suggest that the responses of the knee, pelvis, thorax, and shoulder in the pedestrian model are generally within the boundaries of cadaver test corridors under lateral impact loading. The upper body (head, T1, and T8) trajectories show good agreements with the cadaver data in vehicle-to-pedestrian impact configuration. Overall, the FE pedestrian model developed in the current study could be useful as a valuable tool for a pedestrian head safety study.

## 1. Introduction

Pedestrian is the important part of vulnerable road users, and about 22% of the deaths in road traffic accidents in the world are pedestrians [1]. Accident data shows that 64% fatalities and 43% seriously injured pedestrians suffered from head injuries [2, 3]. Although much effort has been made in the vehicle safety design for pedestrian protection, pedestrians still have a high injury risk when struck by current vehicles [4, 5]. Numerical simulations using human body models provide an effective approach to understand pedestrian head injury mechanisms in vehicle crashes, which is the foundation for pedestrian head protection.

Multibody and finite element (FE) human body models are the main tools for predicting pedestrian head kinematics and injuries in vehicle-to-pedestrian collisions. The former was usually used in analyses of pedestrian head kinematics. For example, literatures [6, 7] investigated pedestrian head kinematics in real-world crashes via accident reconstructions using multibody human body models; Elliott et al. [8] used the multibody modelling method to understand the influences of vehicle impact speed, pedestrian speed, and pedestrian gait on pedestrian head kinematics. However, a detailed analysis of injury biomechanical is not available in their study due to the highly simplification of multibody human body models. Therefore, FE modelling was more commonly used for the prediction of pedestrian head injury biomechanics in vehicle collisions. Accident reconstruction using an isolated FE dummy head model or an isolated FE human head model is a widely used approach for the study of pedestrian head injury biomechanics. Yao et al. [9] reconstructed pedestrian head injuries using an isolated FE human head model to build the relationships between predicted physical parameters and real brain injuries in passenger car-to-pedestrian impacts. The similar approach was also employed in latter studies [10–12] to build a pedestrian head injury risk in real-world crashes. Although these studies were based on the real-world accident scenarios, constrains from the neck have not been considered, while neck constrains have significant influences on head kinematics and injuries [13, 14]. Furthermore, most studies of pedestrian head injury using full body FE models only considered limited impact conditions [15, 16] (e.g., impacts at 40 km/h). However, the variation of pedestrian accident scenarios and their influences on pedestrian head kinematics and injuries were ignored [17]. Therefore, the study of pedestrian head safety using a full body FE model and considering a broad range of impact scenarios is likely to be more valid. However, this kind of studies is still scarce due to the long computing time of the current full body FE models required in expensive simulations. For example, the THUMS (Total Human Model for Safety) Academic Version 4.02 AM50 pedestrian model contains around 2,000,000 elements [18], and a simplified pedestrian model (M50-PS) developed based on the GHBMC (Global Human Body Models Consortium) occupant model still has 827,000 elements [19].

Therefore, the purpose of this study is to develop and validate a computationally efficient FE pedestrian model for future studies of pedestrian head safety. For this purpose, the FE pedestrian model developed in the current study should have a relatively small number of elements and a high biofidelity to predicted pedestrian head kinematics and injuries.

## 2. Materials and Methods

### 2.1. Development of the FE Pedestrian Model for Head Safety (PeMHS)

**Figure 1:**
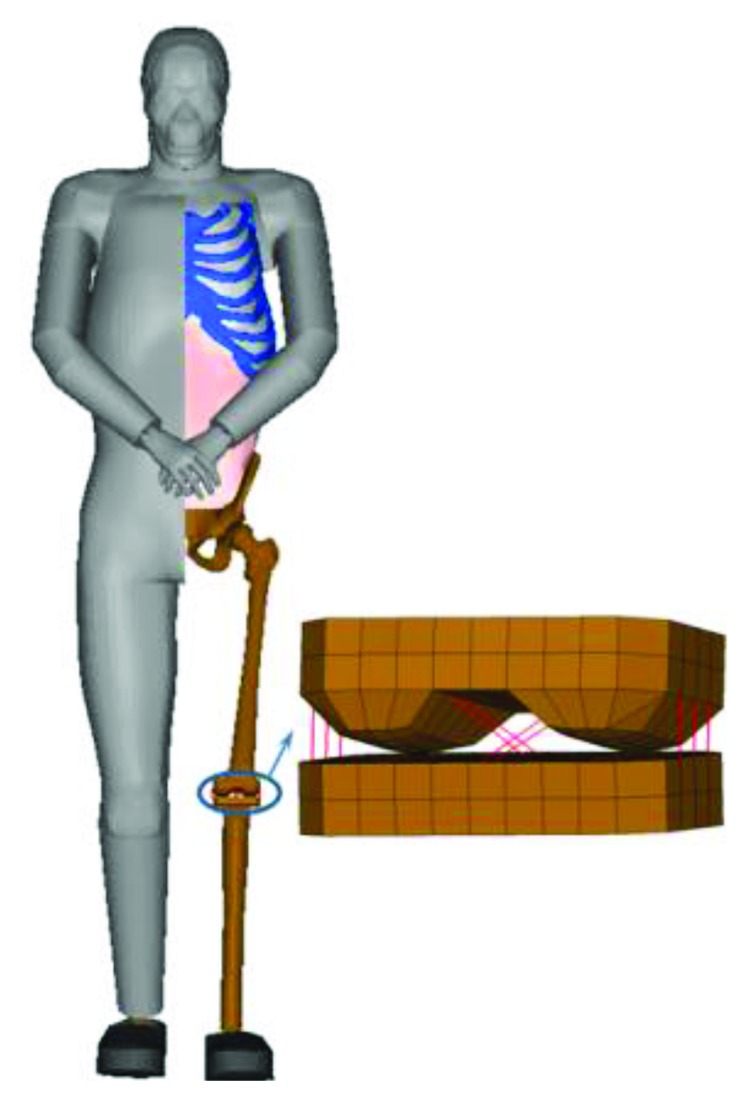
The pedestrian FE model for head safety (PeMHS).

The FE PeMHS model was developed by using LS-DYNA, which includes the head, neck, torso, and upper and lower limbs (see Figure 1). To ensure the biofidelity for predicting head injury, the head and neck models were directly extracted from the THUMS Academic Version 4.02 AM50 (average size male) pedestrian model [18]. The skeletal structures of the torso and pelvis from the THUMS pedestrian model were employed in the current study with these parts being remeshed using lager sized elements. The long bones of the upper and lower limbs were modelled as cylinders with the changes in the cross-section area being considered (Figure 1). Particularly, the femur and long bone in the lower leg (representing the tibia and fibula) was modelled by shell elements with a thickness of 6 mm and 5 mm, respectively. The hip and shoulder joint were developed similar to the THUMS model to keep the physiological characteristics. The bones and ligaments of the knee joint were modelled using simplified geometry and line elements with nonlinear mechanical characteristics, respectively. This approach is similar to that of the FLEX-PLI leg impactor [20] and convenient for gait posture configuration. The ankle was simplified as a spherical joint with constrains by using line elements. The internal organs were modelled as two cavity filling structures for the thoracic and abdominal cavities, respectively, similar to a previous study [19]. The outer flesh parts were modelled using hexahedral elements but with a thin fat layer than the original THUMS model being constructed. In total, the current full body pedestrian FE model contains 173,390 nodes and 432,694 elements.

**Figure 2:**
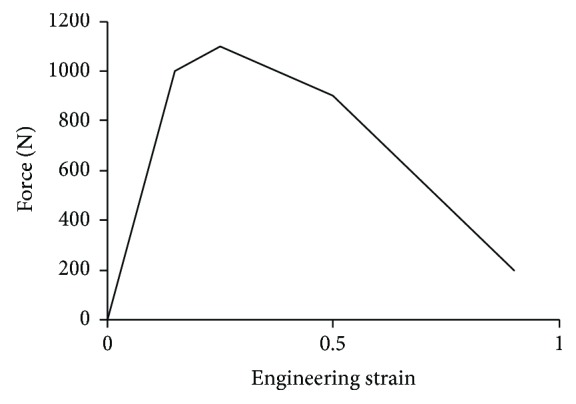
Load curve of the material for knee ligaments.

To ensure a good biofidelity, the same material properties as the THUMS model were employed by the PeMHS for the head, neck, skeletal structures of the torso and pelvis, soft tissues in the spine, shoulder and hip joint, and outer flesh parts. For other simplified body parts, viscoelastic material properties (MAT 06 in LS-DYNA) were defined for the filling parts in thoracic and abdominal cavity, elastic-plastic material properties (MAT 024 in LS-DYNA) were used for the simplified long bones, and seatbelt material properties (MAT B01 in LS-DYNA) with a nonlinear load curve (force vs. engineering strain) were employed for knee ligament. The material parameters for these simplified body parts are given in Table 1, and Figure 2 shows the load curve of the material for knee ligaments. The selection of these material properties was mainly based on previous studies of the development of FE human body models [21–23].

### 2.2. PeMHS Model Validation at the Segment Level

The focuses in model validation at the segment level are on representative body regions which may affect pedestrian head kinematics in vehicle collisions. Therefore, validations on the knee, pelvis, abdomen, thorax, and shoulder were conducted against cadaver test data from the literature, similar to a previous study [19]. The material properties for these body parts of the model were optimized during the validation process to match the cadaver data.

**Figure 3:**
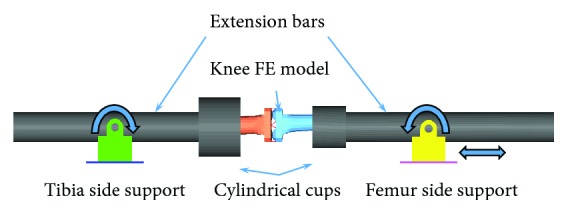
Simulation model for the knee four-point bending test.

**Figure 4:**
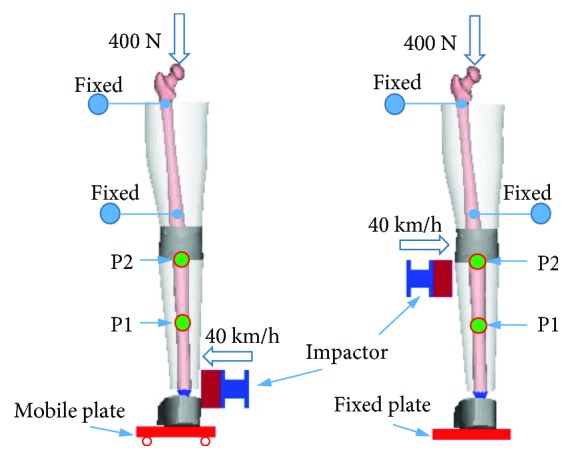
Simulation models for lower limb lateral bending (a) and shearing (b) tests.

Knee lateral sharing and bending are the main kinematics of pedestrians' lower limb in vehicle collisions [24], and then dynamic response of the lower limb may affect pedestrian upper body kinematics [25]. Thus, the lower limb model under lateral impact loading was validated against cadaver knee four-point bending tests conducted by Bose et al. [26] and lateral shearing and bending impact experiments from Kajzer et al. [27], respectively.  Figure 3 shows the simulation model for the knee four-point bending test, where the knee ends were rigidly attached to cylindrical cups of two extension bars. The extension bars were constrained by revolute joints to the corresponding support which was either fully fixed (tibia side) or partially fixed (femur side). A rotational velocity of 1 deg/ms to the knee was defined to simulate the lateral impact of the vehicle-to-pedestrian knee at 40 km/h. The simulation models for lateral bending and shearing impact validation are shown in Figure 4. In the cadaver tests from Kajzer et al. [27], a fixed foot plate was used to represent the normal friction from the ground in bending tests, while the foot was placed on a moveable plate in shearing teats. The proximal of the femur was fixed with screws, while the distal of the femur was fixed with a fixed plate to limit its horizontal movement. A force of 400 N was loaded at the hip to simulate the weight of the upper body. The impact load was conducted at 40 km/h with an impactor of 6.25 kg, where a foam was wrapped at the front to obtain a soft contact. The impact location is at the ankle joint and the knee joint (not contact with the femur condyle) for bending and shearing tests, respectively. Similar to previous studies of pedestrian lower limb model validation [22, 28], displacement from two targets (P1 and P2 in Figure 4) on the tibia was extracted from simulations to compare with the cadaver test data.

**Figure 5:**
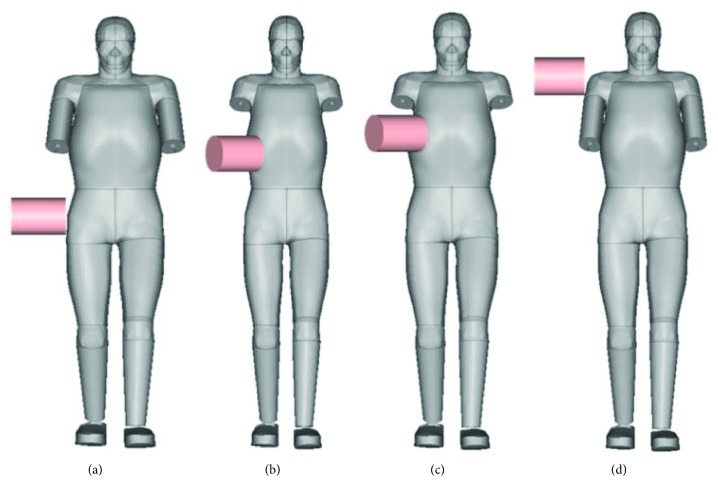
Simulation models for pelvis (a), abdomen (b), thorax (c), and shoulder (d) validation.

The biofidelity of the pelvis, abdomen, thorax, and shoulder regions was validated against cadaver test data under lateral impact loading from previous studies [29, 30].  Figure 5 shows the simulation models for these validation tests, and the corresponding information is summarized in Table 2. For impact tests to the pelvis, abdomen, and thorax, the impactor is 23.4 kg in weight and 150 mm in diameter, while the impactor mass is 23 kg with the same dimension for the shoulder impact test. The impact direction for the pelvis and shoulder tests was defined at the medial-lateral direction, while this was defined as 30° toward the medial-lateral direction for the abdomen and thorax impact tests. The impact location was defined at the level of greater trochanter, 7.5 cm down from the xiphoid process, aligned to the xiphoid process and the shoulder region for the pelvis, abdomen, thorax, and shoulder tests, respectively. In these tests, the impactors were freely suspended and accelerated to the impact speeds and the cadavers were in an upright supported posture with hands and arms overhead to avoid interference between the arm and impactor. In the simulations, the arm (abdomen and thorax validation) or forearm (pelvis and shoulder validation) was removed from the full body model to avoid interference, but the corresponding mass was attached to the adjacent parts (shoulder or elbow) to keep the inertial force. This approach has also been used in a previous study of the validation of a pedestrian human body model [19]. The time history of impact force was calculated for each impact simulation and compared to the corresponding test data to validate the FE model.

### 2.3. PeMHS Model Validation at the Full Body Level

**Figure 6:**
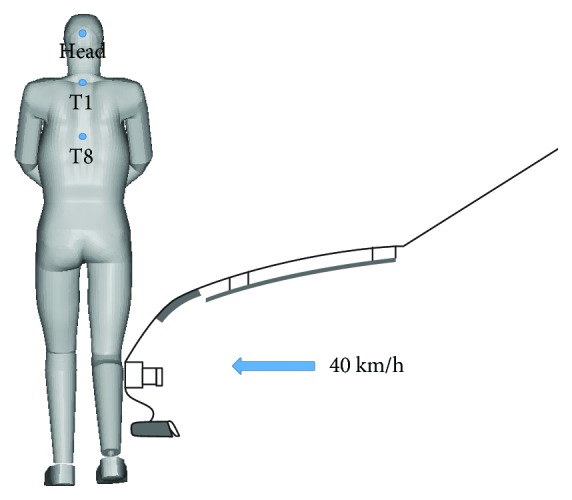
The simulation model for full body validation.

The FE PeMHS model was validated against the vehicle-to-pedestrian impact tests using post mortem human subject (PMHS) from the literature, where specimens with a stature between 170 and 175 cm and a weight between 50 and 85 kg were chosen [25]. A simplified sedan front FE model was developed based on the geometry of the car used in the PMHS tests. This simplification approach of a car front model has been used in previous studies [31, 32].  Figure 6 shows the vehicle-to-pedestrian impact simulation model which was set according to the initial conditions of the PMHS test. In the tests, the hands of the HBMP model were tied in front and the legs were set in a walking posture with the left leg backward and the right leg forward; positioning was achieved with the help of harness straps directed under the arms, which was released prior to impact; the vehicle impact velocity was at 40 km/h from the right sight of the specimens. In the study of Kerrigan et al. [25], boxed corridors based on a percentage of trajectory path length were developed from the PMHS trajectory data. The 10% path length corridors from Kerrigan et al. [25] were used for the validation of the PeMHS upper body trajectories, including the head, T1, and T8. Thus, trajectories of the upper body (head, T1, and T8) were calculated in the simulation according to the corresponding locations of the record mark fixation points in the PMHS tests (see Figure 6). For a further evaluation on the biofidelity of the PeMHS model, the prediction from the PeMHS model was compared with that from the THUMS model under the same impact configuration as shown in Figure 6.

In order to quantitatively assess the correlation between the predictions and cadaver test data, the CORA (correlation and analysis) method was applied. The CORA rating results are within the range from 0% (no correlation) to 100% (perfect match). The CORA has two methods to assess the correlation between signals, where the corridor method (CORA-CD) calculates the deviation between the predicting curve and the reference corridors; the cross correlation method (CORA-CL) evaluates specific curve fitness to the target through parameters such as the phase shift or shape of the signals [33]. Here, an equal weighting was employed for CORA-CD and CORA-CL, i.e., CORA = 0.5^∗^CORA‐CD + 0.5^∗^CORA‐CL.

## 3. Results

### 3.1. Model Validation at the Segment Level

**Figure 7:**
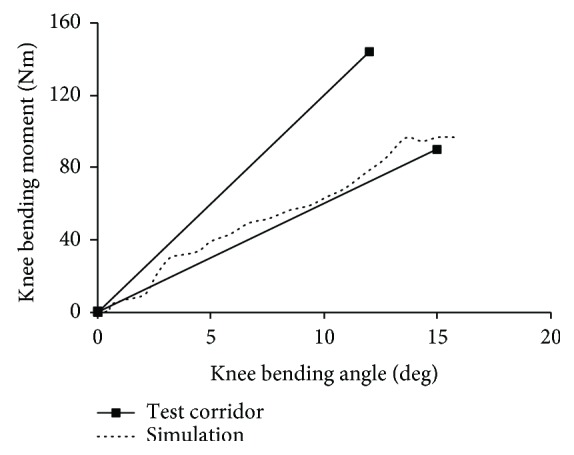
Predicted knee bending moment-bending angle curve versus cadaver test data of knee four-point bending.

**Figure 8:**
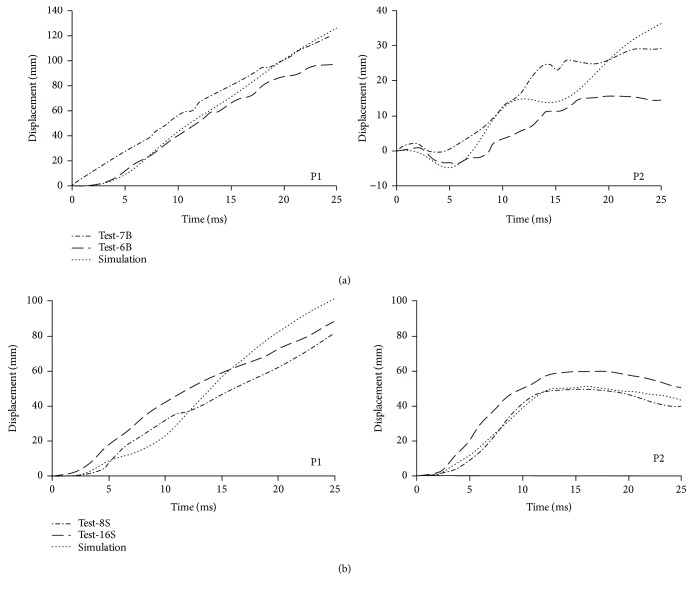
Predicted tibia displacement (P1 and P2) time history versus cadaver test data of lower limb bending (a) and shearing (b).

Figure 7 compares the predicted knee bending moment-bending angle curve with the corridor adapted from cadaver test data of knee four-point bending.  Figure 8 shows the predicted tibia displacement (P1 and P2) time history curves in lower limb bending and shearing impacts together with the cadaver data of Test-7B, Test-6B, Test-8S, and Test-16S from Kajzer et al. [27], for which the height and weight of the sample are relatively close to the PeMHS model.

**Figure 9:**
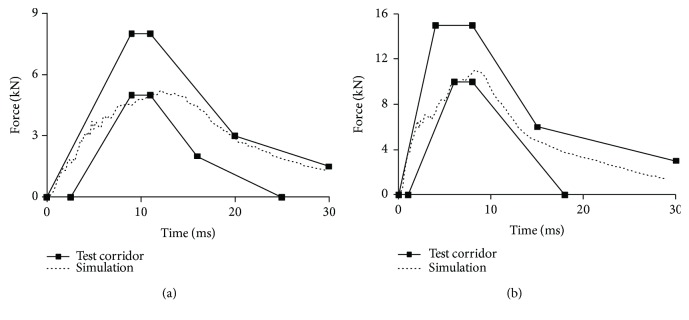
Predicted impact force time history versus cadaver test data in pelvis impact: impact speed = 4.5 m/s (a) and 6.8 m/s (b).

**Figure 10:**
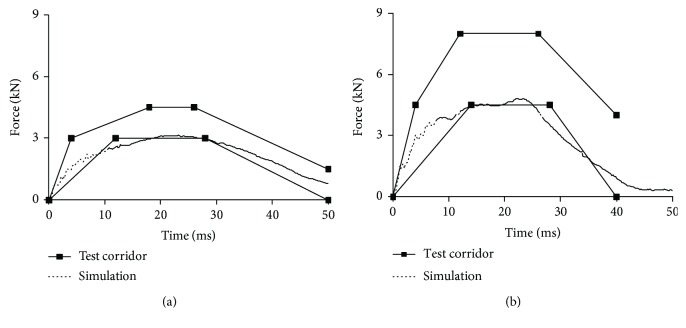
Predicted impact force time history versus cadaver test data in abdomen impact: impact speed = 6.8 m/s (a) and 9.4 m/s (b).

**Figure 11:**
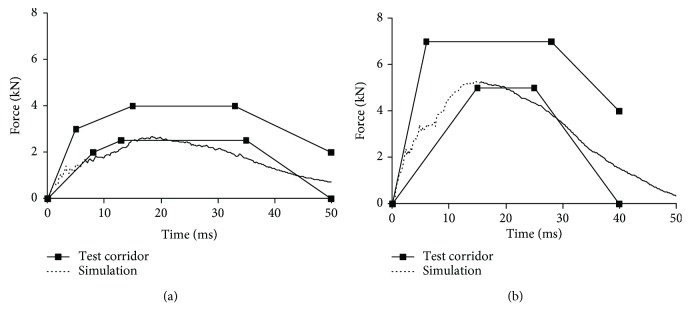
Predicted impact force time history versus cadaver test data in thorax impact: impact speed = 6.5 m/s (a) and 9.8 m/s (b).

**Figure 12:**
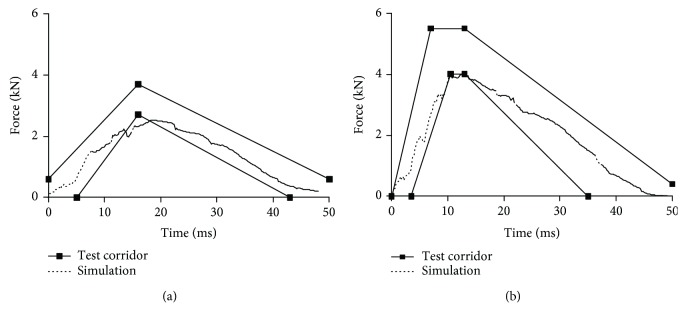
Predicted impact force time history versus cadaver test data in shoulder impact: impact speed = 4.5 m/s (a) and 6.8 m/s (b).

Figures 9–12 show the predicted impact force time history together with the corridor in the corresponding test for pelvis, abdomen, thorax, and shoulder, respectively. Generally, the predicted curves from the simulations are within the cadaver test corridors.

### 3.2. Model Validation at the Full Body Level

**Figure 13:**
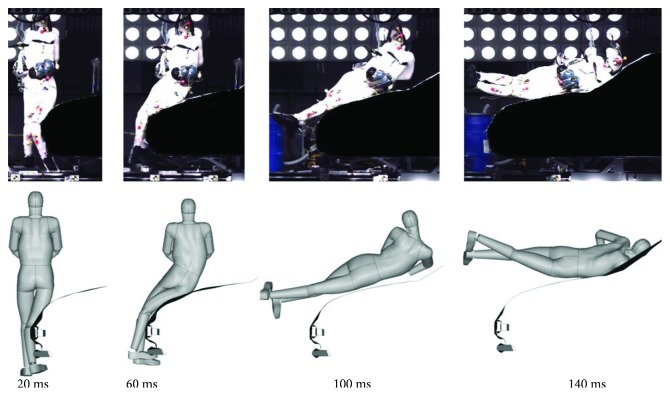
Predicted overall kinematics of the PeMHS model versus cadaver test data in vehicle-to-pedestrian impact.

**Figure 14:**
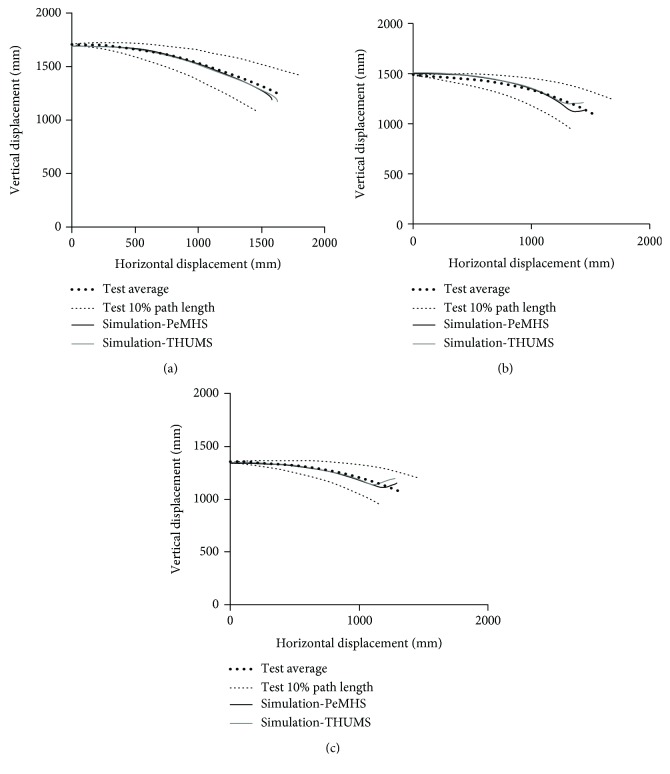
Predicted trajectories of the head (a), T1 (b), and T8 (c) versus cadaver test data in vehicle-to-pedestrian impact.

**Figure 15:**
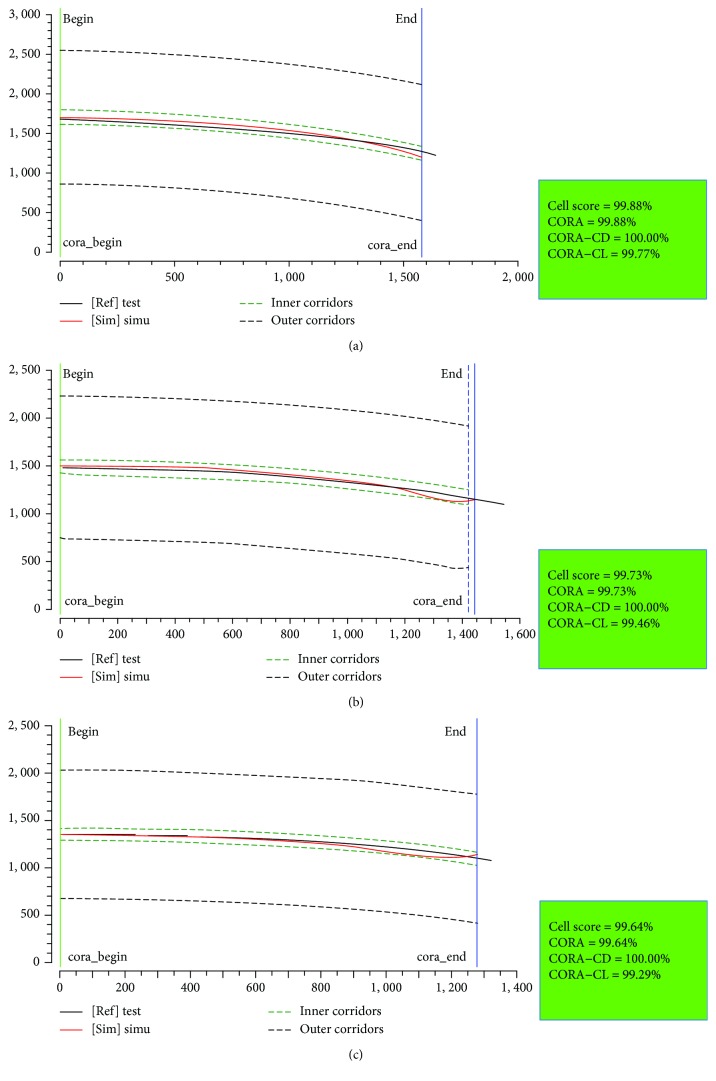
CORA rating results for the trajectories of the head (a), T1 (b), and T8 (c) predicted from the PeMHS model versus cadaver test data in vehicle-to-pedestrian impact.

**Figure 16:**
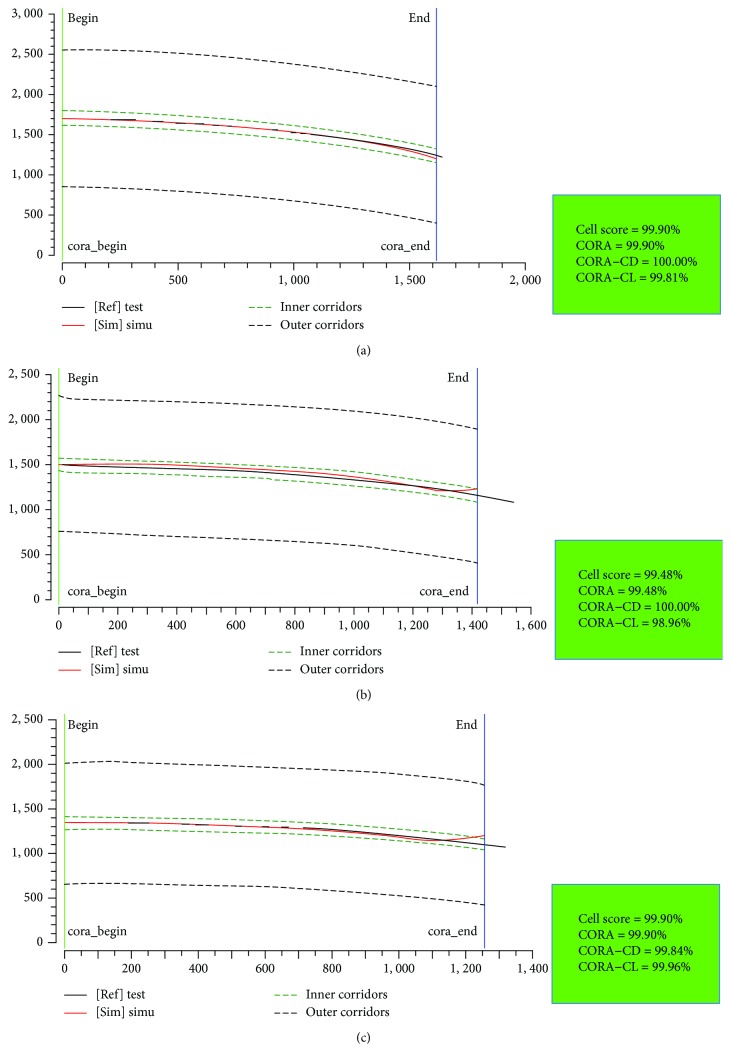
CORA rating results for the trajectories of the head (a), T1 (b), and T8 (c) predicted from the THUMS model versus cadaver test data in vehicle-to-pedestrian impact.

Figure 13 compares the overall pedestrian kinematics between the PeMHS model and cadaver test data from Kerrigan et al. [25]. The predicted overall kinematics of FE pedestrian models is reasonably close to the test data, though some differences are observed in the pelvis and lower limbs for the PeMHS model at the latter stage (>100 ms) and the time of head contact on the windshield. The global pedestrian kinematics and trajectories of the head, T1, and T8 predicted from the PeMHS model are compared with that from the THUMS model and cadaver test data in Figure 14, and the quantitative assessment results referring to the test average data are being summarized in Table 3 (see Figures 15 and 16 for detailed CORA rating data). Overall, the predicted trajectories are similar between the PeMHS model and THUMS model, and both match those of test average well in the initial phases of motion, though there are some differences towards the end of the simulation. Nevertheless, the trajectories of the FE models do always remain within the PMHS test corridors, and the CORA rating results (>99%) are all close to 100% (perfect match).

**Figure 17:**
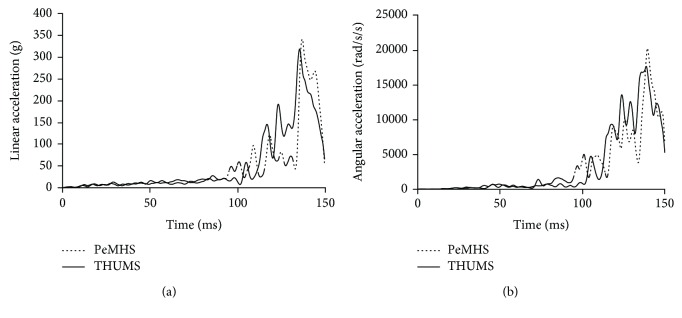
Comparisons of predicted head linear acceleration (a) and angular acceleration (b) between the PeMHS model and THUMS model in vehicle-to-pedestrian impact.

Figure 17 compares the predicted head linear and angular acceleration curves between PeMHS model and THUMS model, respectively. The predictions from the PeMHS model are generally similar to those from the THUMS model as to the curve trend and peak time, though there are some differences in the peak value.

## 4. Discussion

### 4.1. Computational Efficiency

The main purpose of the current study is to develop a computationally efficient full body FE model for pedestrian head kinematics and injury prediction. The original THUMS head models have been previously evaluated to be generally credible for human head injury prediction [32, 34]. Thus, the original head and neck models from the THUMS were employed in the PeMHS model to keep the injury prediction capability for head injuries. To reduce the total element number, the body parts below the neck were redeveloped using simplified geometry (e.g., upper and lower limbs and internal organs) or bigger sized elements (e.g., spine and ribcage). In particular, the element number (432,694) of the PeMHS is only about 25% of the THUMS Academic Version 4.02 AM50 pedestrian model (about 2,000,000 elements [18]) and the original GHBMC model (about 2,100,000 elements [19]) and half of the M50-PS model (about 827,000 elements [19]). The smaller number of elements can improve the computational efficiency of the human body model in simulations, especially when a big number of simulations were needed. According to the theory of FE modelling, using a bigger computing time step and defining some body parts as rigid bodies can improve the computational efficiency of the human body FE model. However, these may affect simulation accuracy and pedestrian kinematics [19]. Thus, the simplification approach used in the current study is logically feasible.

### 4.2. Model Biofidelity

For biofidelity of the PeMHS model, both the validation results at the segment level and full body level show good agreement with cadaver test data (Figures 7–14). Particularly, all predictions at the segment level are in the cadaver test ranges. However, the predictions are mostly close to the lower boundary (Figures 7 and 9–12). Similar results were also observed in a previous study of simplifying a pedestrian model [19]. Many factors could affect model response such as impact boundary conditions, material properties, and thoracic cavity filling approach. Further analysis is still necessary to improve model biofidelity. Nevertheless, the biofidelity of the knee, pelvis, abdomen, thorax, and shoulder of the PeMHS model is generally plausible given the purpose of model use. For the validation at the full body level, the trajectories of the PeMHS model (head, T1, and T8) and cadaver data showed very good agreement, especially at the early stage (<60 ms) of the impact. However, at the latter stage (>100 ms) of the impact, the trajectories of T1 and T8 showed some differences from the cadaver average data and the head contact time is earlier in the simulation (Figures 13 and 14). These discrepancies are probably due to anthropometric differences, uncertainty with respect to the initial position of the PMHS, simplification of the lower limb anatomical structure, and model tissue level properties (such as knee ligaments and internal organs). It seems that the lower limb of the PeMHS is kind of too stiffer since an excessive rebound was observed in the leg and pelvis (Figure 13). This is largely due to the properties of the long bones in the model; further improvement to these parts is still needed. Kinematics differences between the FE model and cadaver data could also be found in the original validation [35] and further evaluation of THUMS [32] and validation of the M50-PS model [19]. This highlights challenges in validating pedestrian human body models against PMHS data when full details of the cadaver characteristics are not exactly the same as the models. Nevertheless, the predicted pedestrian upper body trajectories are within the cadaver corridors and close to the cadaver average value (CORA rating scores > 99%). The comparisons between the PeMHS model and THUMS model also showed good agreements for both upper body trajectories (Figure 14) and head accelerations (Figure 17). This also implies that the model developed in this study is potentially capable of predicting pedestrian head kinematics and injuries in vehicle collisions, given the recognized biofidelity of the THUMS model [32, 34, 36, 37].

### 4.3. Limitations

There are some limitations in this study. More precision treatment is still needed for the simplification approach to the anatomical structure of human body parts, and the material properties for these simplified body regions, though the predictions of the PeMHS, are generally comparable to cadaver test data and predictions of the THUMS model. Only a single vehicle model and the lateral impact scenario were considered in the validation process due to limited availability of test data; a broad range of impact scenarios should be considered in further evaluations to prove the PeMHS model as a robust tool for a pedestrian head safety study. However, this is one of the common difficulties in FE human body evaluation [19, 32, 36, 37], and the lateral impact configuration is dominant in real-world vehicle-to-pedestrian accidents [38].

## 5. Conclusions

The current study developed and validated a computationally efficient FE pedestrian model for head safety analysis. The FE pedestrian model developed in this study has a small number of elements, which can ensure the computational efficiency. The validation results indicate a good capability of this model in predicting pedestrian dynamic responses at both segment and full body levels. Therefore, the current FE pedestrian model could be useful as a valuable tool for future research of pedestrian head safety in vehicle collisions.

## Figures and Tables

**(a):** 

	Material model	Material parameters			
Tissues	Elastic-plastic	*ρ* (kg/m^3^)	*E* (MPa)	*δ* (MPa)	Pr
Femur		2,080	13,500	115	0.3
Tibia		1,900	20,033	125	0.315

**(b):** 

	Material model	Material parameters				
Tissues	Viscoelastic	*ρ* (kg/m^3^)	*k* (MPa)	*G*_0_ (kPa)	*G*_*i*_ (kPa)	*β*
Thoracic cavity		1,000	4.5	7.15	4.15	0.25
Abdominal cavity		1,000	0.25	54	40	0.25

*ρ*: density; *E*: Young's modulus; *δ:* yield stress; Pr: Poisson's ratio; *k*: Bulk modulus; *G*_0_: short-term shear modulus; *G*_*i*_: long-term shear modulus; *β*: decay constant.

**Table 2:** Information of impact conditions for pelvis, abdomen, thorax, and shoulder validation.

Segment	Impact speed	Impact direction	Impact location
Pelvis	5.2 and 9.8 m/s	Medial-lateral direction	Greater trochanter
Abdomen	6.8 and 9.4 m/s	30° toward the medial-lateral direction	7.5 cm below the xiphoid process
Thorax	6.5 and 9.5 m/s	30° toward the medial-lateral direction	Aligned to the xiphoid process
Shoulder	4.5 and 6.8 m/s	Medial-lateral direction	Shoulder region

**Table 3:** CORA rating results for trajectories of the head, T1, and T8.

Signal	PeMHS			THUMS		
	CORA-CD	CORA-CL	CORA	CORA-CD	CORA-CL	CORA
Head	100%	99.77%	99.88%	100%	99.81%	99.90%
T1	100%	99.46%	99.73%	100%	98.96%	99.48%
T8	100%	99.29%	99.64%	99.84%	99.96%	99.90%

## Data Availability

The data used to support the findings of this study are included within the article.
